# The second generation of HIV-1 vertically exposed infants: a case series from the Italian Register for paediatric HIV infection

**DOI:** 10.1186/1471-2334-14-277

**Published:** 2014-05-20

**Authors:** Carmelina Calitri, Clara Gabiano, Luisa Galli, Elena Chiappini, Carlo Giaquinto, Wilma Buffolano, Orazio Genovese, Susanna Esposito, Stefania Bernardi, Maurizio De Martino, Pier-Angelo Tovo

**Affiliations:** 1 Department of Paediatrics, University of Turin, Turin, Italy; 2 Department of Health Sciences, University of Florence, Florence, Italy; 3 Department of Paediatrics, Padua University, Padua, Italy; 4 Coordinating Centre for Perinatal Infection of Campania Region, Translational Medical Sciences Department of Federico II University, Naples, Italy; 5 Department of Emergency, Catholic University of Rome, Rome, Italy; 6 Paediatric Clinic 1, Department of Pathophysiology and Transplantation, Fondazione IRCCS Ca’ Granda Ospedale Maggiore Policlinico, Milan, Italy; 7 Department of Immunology and Infectious Diseases, "Bambino Gesù" Children’s Hospital, Rome, Italy

**Keywords:** HIV-1, Drug-resistant virus, AZT, Vertical transmission

## Abstract

**Background:**

In the Highly Active Antiretroviral Therapy (HAART) era, the prognosis of children perinatally infected with HIV-1 has significantly improved, so the number of perinatally-infected females entering child-bearing age and experiencing motherhood is increasing.

**Methods:**

A description of the medical history and pregnancy outcomes of women with perinatal acquired HIV-1 infection enrolled in the Italian Register for HIV infection in Children.

**Results:**

Twenty-three women had 29 pregnancies. They had started an antiretroviral therapy at a median of 7.7 years (interquartile range, IQR 2.3 - 11.4), and had experienced a median of 4 therapeutic regimens (IQR 2–6). Twenty women (87%) had taken zidovudine (AZT) before pregnancy, in 14 cases as a starting monotherapy. In 21 pregnancies a protease inhibitor-based regimen was used. At delivery, the median of CD4+ T lymphocytes was 450/μL (IQR 275–522), and no viral load was detectable in 15 cases (reported in 21 pregnancies). Twenty-eight children were delivered through caesarean section (median gestational age: 38 weeks, IQR 36–38, median birth weight: 2550 grams, IQR 2270 – 3000). Intravenous AZT was administered during delivery in 26 cases. All children received oral AZT (median: 42 days, IQR 31 – 42), with no adverse events reported. No child acquired HIV-1 infection.

**Conclusions:**

Despite a long history of maternal infection, multiple antiretroviral regimens and, perhaps, the development of drug-resistant viruses, the risk of mother-to-child transmission does not seem to have increased among the second-generation of HIV-1 exposed infants.

## Background

The introduction of Highly Active Antiretroviral Therapy (HAART) has significantly improved the quality of life and prognosis of children with perinatally acquired HIV-1 infection in resource-rich countries [1,2]. As a result, an increasing number of these children are entering adolescence and young adulthood, with a consequent proportion of females becoming sexually active and pregnant. These mothers presented with a long history of HIV-1 infection and sometimes AIDS complications. They had a wide exposition to antiretroviral (ART) drugs and to their, primarily metabolic, chronic side effects. They experienced multiple ART regimens, which were often sub-therapeutic until the advent of HAART, with a consequent risk of acquiring drug-resistant viruses.

Therefore, it has since become crucial to define the pregnancy outcome and effectiveness of ART therapy in preventing mother-to-child HIV-1 transmission (MTCT) in this 2nd generation of HIV-1 exposed infants. Reports from Europe [3,4], Puerto Rico [5], India [6], USA [7-10], and Brazil [11] describe favourable maternal and neonatal outcomes, with a MTCT rate varying from 0 to 7.7%, detected in cases of poor adherence to therapy [10].

Here, we describe the clinical aspects of pregnancy in women with perinatally acquired HIV-1 infection enrolled over the years in the Italian Register for HIV Infection in Children.

## Methods

The Italian Register for HIV infection in children (ITLR) is a nationwide, multicentre, prospective study set up by the Italian Association of Paediatrics in 1985. At the moment of patient’s enrolment, written informed consent is obtained from the patient’s guardians; data are treated anonymously as each patient is identified by an alphanumerical code. The study was approved by the review boards and ethics committees of each participating institution, as described in detail elsewhere [1,12]. A retrospective analysis of perinatally HIV-1 infected females, enrolled in the ITLR from its institution and who had a child, was carried out. Information included: the women’s demographic and clinical characteristics, such as their mothers’ risk factors for HIV-1 infection, perinatal ART use, HIV-1 related diseases and comorbidities; the number and types of ART regimens both before and during pregnancy; age at delivery, number of CD4+ T-lymphocytes and viral load at delivery, the mode of delivery; infants’ characteristics (gestational age, sex, birth weight, congenital malformations, any other acute or chronic diseases, adverse events for MTCT prophylaxis, HIV-1 infection status). SPSS (version 20.0) software for Windows was used for data management.

## Results

Between 2001 and 2012, 29 children were born to 23 women with perinatal HIV-1 infection. One woman had 3 pregnancies and four women had 2 pregnancies each. See Tables 1, 2 and 3 for data concerning mother-child pairs.

**Table 1:** Characteristics of HIV-1 vertically infected women

**N.**	**Birth order**	**Risk factors for HIV infection in grandmothers**	**Year of birth**	**Age (years) at start of ART therapy**	**AZT monotherapy**	**No of regimens experienced**	**Years of ART therapy (before the first pregnancy)**	**Clinical and immunological category at last observation**	**Age (years) at AIDS onset**	**Age (years) at delivery**
Case 1	1^st^	Drug abuse	1982	14.6	No	4	13.2	A 1		28.4
Case 2	1^st^	Sexual intercourse with drug abuser, Drug abuse	1982	10.6	Yes	5	7.7	B 3		24.6
	2^nd^									26.9
Case 3	1^st^	Risk sexual intercourse	1983	7.7	Yes	6	17.5	B 3		26.1
Case 4	1^st^	Drug abuse	1983	11.4	Yes	7	7	B 3		23.9
Case 5	1^st^	Unknown	1983	21.7	No	1	0	B 3		22
	2^nd^									23.7
	3^th^									24.7
Case 6	^1st^	Drug abuse	1984	6.6	Yes	6	9.7	B 2		17
	2^nd^									18.3
Case 7	1^st^	Sexual intercourse with drug abuser	1985	3.1	No	2	20.5	N 2		24.3
Case 8	1^st^	Drug abuse	1985	3	Yes	15	17.7	C 3	11.5	22.4
Case 9	1^st^	Sexual intercourse with drug abuser, Drug abuse	1986	12.2	No	2	9.5	C 2	13.1	22.2
Case 10	1^st^	Sexual intercourse with drug abuser	1986	6.6	Yes	6	14.8	C 3	6.6	22.1
Case 11	^1st^	Unknown	1986	10.2	No	6	11.1	A 3		22
Case 12	1^st^	Sexual intercourse with drug abuser	1986	10	Yes	3	6.3	A 2		21.1
	2^nd^									22.2
Case 13	1^st^	Unknown	1986	13.3	No	1	11	A 1		25
Case 14	1^st^	Sexual intercourse with drug abuser	1987	2.3	Yes	3	21.6	B 2		24.5
Case 15	^1st^	Unknown	1988	14.4	No	2	10.3	B 1		22.6
Case 16	1^st^	Unknown	1988	1.9	Yes	2	6.2	A 2		19.2
Case 17	1^st^	Sexual intercourse with drug abuser	1989	1.8	Yes	5	18	A 1		20.3
Case 18	1^st^	Sexual intercourse with drug abuser, Drug abuse	1990	1.7	Yes	5	14.4	B 2		20.1
Case 19	1^st^	Drug abuse	1990	5.4	Yes	8	13.9	B 3		20
Case 20	^1st^	Unknown	1990	8.6	No	4	11.6	A 3		20.8
	2^nd^									21.7
Case 21	1^st^	Sexual intercourse with drug abuser	1991	1.2	Yes	3	17.3	B 1		19.3
Case 22	1^st^	Drug abuse	1991	0.6	Yes	7	19.2	C 3	0.2	20.4
Case 23	1^st^	Sexual intercourse with drug abuser, Drug abuse	1991	8.2	No	2	9.9	A 2		20

Legend. ART = antiretroviral; AZT = zidovudine.

**Table 2:** Pregnancy features

**N.**	**Birth order**	**ART therapy at conception**	**ART therapy interruption at first trimester**	**ART regimen during pregnancy**	**Gestational week at start**	**Gestational week at end**	**CD4+ at delivery**	**VL at delivery**	**AZT intrapartum**	**Type of delivery**
Case 1	1^st^	No	No	AZT + 3TC + ATV/rtv	5	37	525	Undetectable	Yes	EC
Case 2	1^st^	Yes	Yes (from 5^th^ to 12^th^ week)	DDI + 3TC + NVP	1	4	480	157	Yes	EC
				AZT + 3TC + NVP	13	38				
	2^nd^	Yes	No	FTC + TDF + FPV/rtv	1	38	520	Undetectable	Yes	EC
Case 3	1^st^	Yes	No	FTC + TDF + ATV/rtv	1	38	125	Undetectable	Yes	EC
Case 4	1^st^	No	No	No			Unknown	Unknown	Yes	EC
Case 5	1^st^	No	No	AZT + 3TC + LPV/rtv	23	34	307	Unknown	Yes	EC
	2^nd^	Yes	No	AZT + 3TC + LPV/rtv	1	37	480	15332	Yes	EC
	3^th^	Yes	No	AZT + 3TC + LPV/rtv	1	Unknown	Unknown	Unknown	Yes	EC
Case 6	1^st^	No	No	AZT + 3TC + NVP	6	36	Unknown	Unknown	No	EC
	2^nd^	No	No	AZT + 3TC + NVP	6	40	Unknown	Unknown	No	EC
Case 7	1^st^	Yes	No	ABC + 3TC + ATV	1	34	323	Undetectable	Yes	EC
				ABC + 3TC + ATV/rtv	35	38				
Case 8	1^st^	Yes	No	FTC + TDF + DRV/rtv	1	38	450	Undetectable	Yes	EC
Case 9	1^st^	Yes	No	ABC + DDI + LPV/rtv	1	16	312	7653	Yes	EC
				AZT + 3TC + LPV/rtv	17	36				
Case 10	1^st^	Yes	Yes (from 6^th^ to 12^th^ week)	FTC + TDF + EFV	1	5	240	Undetectable	Yes	EC
				AZT + 3TC + LPV/rtv	13	38				
Case 11	1^st^	Unknown	No	ABC + TDF + LPV/rtv	Unknown	39	Unknown	Undetectable	Yes	EC
Case 12	1^st^	No	No	No			Unknown	Unknown	Yes	UC
	2^nd^	Yes	No	AZT + 3TC + LPV/rtv	1	38	95	Unknown	Yes	EC
Case 13	1^st^	Yes	No	FTC + NVP	1	39	673	Undetectable	Yes	EC
Case 14	1^st^	Yes	No	AZT + 3TC + ABC	1	35	564	Undetectable	Yes	EC
Case 15	1^st^	Yes	No	FTC + TDF + DRV/rtv	1	37	501	Undetectable	Yes	EC
Case 16	1^st^	No	No	FTC + TDF + ATV/rtv	16	36	522	Undetectable	Yes	EC
Case 17	1^st^	No	No	FTC + TDF + ATV	13	37	770	Undetectable	Yes	EC
Case 18	1^st^	Yes	No	3TC	1	28	440	13099	Yes	LC
				FTC + TDF + DRV/rtv	29	36				
Case 19	1^st^	No	No	FTC + TDF + ATV/rtv	6	38	501	Undetectable	Yes	EC
Case 20	1^st^	No	No	FTC + TDF + DRV/rtv + RAL	38	40	200	Undetectable	Yes	EC
	2^nd^	Unknown	Unknown	Unknown			99	Unknown	Unknown	Unknown
Case 21	1^st^	Yes	No	FTC + TDF + ATV/rtv	1	38	776	615	Yes	EC
Case 22	1^st^	Yes	No	AZT + DDI + LPV/rtv	1	35	435	Undetectable	Yes	EC
Case 23	1^st^	Yes	Yes (from 3^rd^ to 9^th^ week)	FTC + TDF + LPV/rtv	1	3	275	20000	Yes	EC
				FTC + TDF + LPV/rtv	10	38				

Legend. VL: viral load (copies/ml); AZT: zidovudine; 3TC: lamivudine; ABC: abacavir; FTC: emtricitabine; TDF: tenofovir; DDI: didanosine; NVP: nevirapine; LPV: lopinavir; ATV: atazanavir; FPV: fosamprenavir; DRV: darunavir; rtv: ritonavir booster; RAL: raltegravir; EC = elective caesarean section; UC = unspecified caesarean section; LC = caesarean section after labour.

**Table 3:** HIV-1 exposed children features

**N.**	**Birth order**	**Year of birth**	**Gender**	**Gestational age (weeks)**	**Weight at birth (grams)**		**ART prophylaxis**	**Duration of postnatal ART prophylaxis (days)**	**HIV-1 status**	**Other pathological conditions**
Case 1	1^st^	2010	M	37	2590		AZT	42	SR	
Case 2	1^st^	2007	F	38	2550		AZT	45	SR	
	2^nd^	2009	M	38	3240		AZT	19	SR	
Case 3	1^st^	2009	F	38	3100		AZT	28	SR	
Case 4	1^st^	2007	F	38	2920		AZT	36	SR	
Case 5	1^st^	2005	F	34	1975		AZT	42	SR	
	2^nd^	2007	F	37	1960	SGA	AZT	42	SR	HCV infection
	3^th^	2008	F	Unknown	Unknown		AZT	28	SR	
Case 6	1^st^	2001	F	36	1880	SGA	AZT	44	SR	
	2^nd^	2002	F	40	3080		AZT	42	SR	
Case 7	1^st^	2009	M	38	2530		AZT	42	SR	
Case 8	1^st^	2008	F	38	2450		AZT	43	SR	
Case 9	1^st^	2008	F	36	2520		AZT	42	SR	
Case 10	1^st^	2008	M	38	3250		AZT	31	SR	
Case 11	1^st^	2008	F	39	2770		AZT	41	SR	
Case 12	1^st^	2007	M	35	2270		AZT + 3TC + NVP	31	SR	
	2^nd^	2008	M	38	3000		AZT + 3TC + NVP	42	SR	
Case 13	1^st^	2011	M	39	3100		AZT	41	SR	
Case 14	1^st^	2011	F	35	1190	SGA	AZT	18	SR	
Case 15	1^st^	2011	M	37	3190		AZT	42	SR	
Case 16	1^st^	2008	F	36	2760		AZT	42	SR	
Case 17	1^st^	2009	M	37	2520		AZT	29	SR	Cryptorchidism
Case 18	1^st^	2010	M	36	2180		AZT	42	SR	
Case 19	1^st^	2010	F	38	2500		AZT	14	SR	
Case 20	1^st^	2011	F	40	2380	SGA	AZT	45	SR	
	2^nd^	2012	F	Unknown	Unknown		Unknown	Unknown	SR	
Case 21	1^st^	2010	F	38	2750		AZT	42	SR	
Case 22	1^st^	2012	M	35	2160		AZT	42	SR	
Case 23	1^st^	2011	M	38	2690		AZT	33	SR	

Legend. M = male; F = female; AZT = zidovudine; 3TC = lamivudine; NVP = nevirapine; SR = seroreverter; SGA= small for gestational age.

The median maternal age at first delivery was 22 years (interquartile range (IQR) 20.1-24.3); the oldest mother was born in 1982, the youngest in 1991. No infected mother was perinatally exposed to antiretroviral drugs, as ART prophylaxis was not used at that time. The grandmothers’ risk factors for HIV-1 infection were known for 17 women: 11 had had high risk sexual intercourse and 6 had been IV drug users. All but one woman had ART therapy during childhood or adolescence. The median age at the start of ART therapy was 7.7 years (IQR 2.3-11.4). The median number of regimens for each woman was 4 (IQR 2–6). Twenty (87%) women were given zidovudine (AZT), in 14 cases as a starting monotherapy for a median period of 4.4 years (IQR 2.0-5.2); 12/14 patients were later shifted to a dual therapy containing AZT (median period 2.4 years, IQR 1.0-3.4). All but 2 women received HAART before pregnancy, with a combination of 3 or more drugs, containing at least one protease-inhibitor (PI) in 16 cases. The median time of the mothers’ exposure to ART drugs before their first pregnancy was 11.6 years (IQR 9.5-17.5). Four women developed an AIDS defining condition (at a median age of 9.0 years, IQR 1.8-12.7); 10 women were classified, according to the CDC paediatric classification, in clinical category B, 8 in category A, 1 in category N. As far as the immunological status is concerned, 5 were in category 1, 8 in category 2, and 10 in category 3.

Ten mothers did not have any ART therapy at the time of conception; of the ten, 2 continued without therapy throughout their pregnancy, 4 started ART therapy in the first trimester and 4 after the 12^th^ week of gestation, including one woman who started a rescue therapy including the integrase inhibitor raltegravir at the 38^th^ week of gestation. In the other pregnancies, the women were already undergoing ART treatment at conception, and 3 of them discontinued therapy in the first trimester (see Table 2). In one case, efavirenz (EFV) was continued during the first 5 weeks of gestation, with a switch to a lopinavir/ritonavir containing combination after the 12^th^ week. A PI-based regimen was used in a total of 21 pregnancies. No complications related to pregnancy were noticed. There was a median of 450/μL (IQR 275–522)CD4+ T lymphocytes at the last check before delivery (reported in 23 of the 29 pregnancies); all women with a previous AIDS-defining condition had > 200 CD4+/μL. The viral load at delivery (reported in 21 pregnancies) was undetectable in 15 cases. Intravenous AZT was administered during 26 (89.6%) deliveries. Twenty-eight children were born through caesarean section, which was elective in 26 cases. No neonatal complications for children at birth were reported; 17 (58.6%) were females; their median gestational age was 38 weeks (IQR 36–38) and the median birth weight was 2550 grams (IQR 2270–3000). Eight were preterm (<37 weeks of gestation), and 4 were small for the gestational age (SGA) (Table 3) [13]. No infant was breastfed. Twenty-six infants received oral AZT as MTCT prophylaxis; 2 brothers received a combined regimen (AZT + 3TC + NVP single dose): the first child because of lack of maternal ART therapy during pregnancy, the second one because of severe maternal immunosuppression (CD4+ T-lymphocytes at labour: 95 cell/ μL). Neonatal ART prophylaxis was administered for a median period of 42 days (IQR 31–42) with no major adverse events.

No child acquired HIV-1 infection. One patient was HCV infected and one presented cryptorchidism. The median age at the last check was 3 years (IQR 1.3-4.3), with nine children followed over 4 years of age.

## Discussion

There are few studies exploring the pregnancy outcome in HIV-1 perinatally-infected women. Despite the limited number of pregnancies enrolled and limited amount of available data, ours is one of the largest cohorts with detailed information on the entire maternal history.

The results are reassuring, since none of the 29 exposed infants acquired the infection. Indeed, of the 134 children born to HIV-1 perinatally-infected mothers described in this and other studies [3-11], only 2 acquired the infection, for a total MTCT rate of 1.5%, comparable to that observed in the first-generation of exposed infants with the adoption of the same preventive measures, such as ART therapy during pregnancy, elective caesarean section, ART prophylaxis in the child, and bottle-feeding [12,14,15].

Among 601 women with perinatally acquired HIV-1 infection enrolled in the ITLR, 383 are alive and over 16 years old at the last check. Thus, the 23 described in this case-series represent 6% of those with a similar child-bearing age. It is worth noting that 5 had more than one child. In spite of the prolonged history of HIV-1 infection and of targeted therapies, the good quality of life and a longer life expectancy presumably allowed these women to live motherhood with fewer distressing concerns. The low probability of HIV-1 transmission to the offspring is an additional important factor supporting such women having children. We have no documentation about the possible birth control methods adopted by these women, or whether these pregnancies were planned. However, the median age of our parturients was rather lower than the 32.3 years observed in the general Italian population [16]. This low median age at delivery is consistent with other reports [3,5,6,10], and, perhaps, mirrors the fear that the chronic disease could deprive them of the special female role of motherhood. This growing desire of motherhood among seropositive women clearly emerges from our Register, where the number of HIV-1 perinatally-exposed children is around 350–400 per year, with an increasing multiparity rate, reaching 25% in recent years (data not shown).

In this scenario, the management of HIV-1 perinatally-infected pregnant women and their children will be an important challenge for clinicians in the near future. A crucial aspect will be the choice of the best ART regimen to prevent MTCT. None of our mothers was perinatally exposed to ART drugs, as they were born before the ACTG 076 protocol [17,18]. However, the majority received ART therapy during childhood and experienced several sub-therapeutic regimens. Although resistance data were not available, these conditions are well known to favour the development of drug resistant viruses, with a consequent possible higher risk of MTCT, a phenomenon that fortunately does not emerge from the available data. As far as AZT is concerned, it is worth noting that over half of our mothers started AZT as a monotherapy during childhood, then shifted to a dual therapy including AZT, continuing with such combinations for several years. Six of these 14 women continued to receive AZT also during pregnancy, as did all their children as a prophylaxis, apparently with success, since the majority of the mothers had undetectable viral loads at delivery and no child acquired the infection. This casts doubts on the suggested exclusion of AZT during pregnancy if there had been a prolonged monotherapy during childhood [10]. Indeed, in a recent case series the role of intrapartum intravenous AZT for the prevention of MTCT in women with virological failure has been stressed [19]. AZT may be effective in the prevention of MTCT through other mechanisms besides its direct antiviral effect [18,20].

The possible impact of a prolonged ART therapy on maternal and child health is a further important aspect. Even in this context, our results are reassuring. No congenital abnormalities were noticed, including the child exposed to EFV in the first 5 weeks of gestation [21,22]. Since maternal and neonatal complications due to ART drugs do not seem to be higher in perinatally-infected women, indications should not differ from those commonly recommended in seropositive mothers, for instance HAART should start as soon as possible [15], while stopping ART during pregnancy should be discouraged [12]. In fact, fearing a possible teratogenicity, 3 women discontinued ART treatment during the first trimester of pregnancy and their viral load was detectable at delivery. On the other hand, the experience of several therapeutic regimens during childhood and adolescence will presumably lead to the use of new antiretroviral drugs during pregnancy, such as integrase inhibitors, the impact of which on pregnant women and their foetuses still needs to be elucidated.

## Conclusions

The outcome of second-generation HIV-1 exposed infants seems favourable. In spite of a long course of maternal infection, multiple ART regimens, and a possible development of drug-resistant viral strains, MTCT continues to be efficiently controlled by the commonly adopted preventive strategies, even though an adequate surveillance of pregnancy among this unique population is highly recommended.

## Competing interests

The Italian Register for HIV Infection in Children was funded by the Istituto Superiore di Sanità, Rome, Italy, and the Paediatric European Network for Treatment of AIDS (PENTA) Foundation, Padua, Italy. The authors declare that they have no competing interests.

## Authors’ contributions

All Authors and Co-Authors contributed equally to the study design, acquisition of women and children information and interpretation of data. CC, CG and PAT drafted the manuscript, while MdM, LG, EC, CG, WB, OG, SE, SB critically revised it for its intellectual content. All Authors and Co-Authors accepted the final version of the manuscript.

## Authors’ information

Co-Authors: Giacomo Faldella (Bologna), Raffaele Badolato, Chiara Monfredini (Brescia), Cristina Gotta (Genoa), Vania Giacomet (Milan), Monica Cellini (Modena), Osvalda Rampon (Padua), Maura Agnese (Naples), Piero Valentini (Rome), Carlo Scolfaro, Silvia Garazzino (Turin), Antonio Mazza (Trento).

## Pre-publication history

The pre-publication history for this paper can be accessed here:

http://www.biomedcentral.com/1471-2334/14/277/prepub
